# Supplementation of *Lactobacillus casei* reduces the mortality of *Bombyx mori* larvae challenged by *Nosema bombycis*

**DOI:** 10.1186/s13104-021-05807-1

**Published:** 2021-10-26

**Authors:** Siripuk Suraporn, Olle Terenius

**Affiliations:** 1 Department of Biology, Faculty of Science, Mahasarakham University, Tambon Khamriang, Kantaravichai District, Maha Sarakham, 44150 Thailand; 2 Department of Cell and Molecular Biology, Uppsala University, Box 549, 751 24 Uppsala, Sweden

**Keywords:** Pebrine, Probiotic bacteria, *Lactobacillus casei*, Polyvoltine silkmoth, DokBua

## Abstract

**Objective:**

Pebrine, caused by the microsporidium *Nosema bombycis*, is one of the severe diseases in Thai polyvoltine strains of the silkworm *Bombyx mori*. Studies showing the presence of *Lactobacillus* species in the silkworm gut, where the *Nosema* parasites enter, suggests that these bacteria may have a protective effect. The aim of this study was to investigate the effect of supplementation of *Lactobacillus casei* on the survival ratio of silkworm larvae challenged with *N. bombycis*.

**Results:**

A group of silkworm larvae of the commercial Thai polyvoltine hybrid strain DokBua was supplemented with *L*. *casei* on the second day of the 2nd, 3rd, 4th, and 5th instar. When a control group of silkworm larvae were challenged with *N. bombycis* on the second day of the 4th instar, the survival rate was 68%, but it was 91% for larvae supplemented with *L. casei*. For those larvae that survived the treatments until pupation, we determined the growth characters larval weight, cocooning ratio, and pupation ratio, and the economic characters cocoon weight and cocoon shell weight. When infected with *N. Bombycis*, growth characters were significantly higher in larvae also receiving *L. casei*.

## Introduction

The microsporidium *Nosema bombycis* is an obligate intracellular parasite that causes pebrine disease in the silkworm *Bombyx mori*. *N. bombycis* can be transmitted both horizontally and vertically [1, 2]. In horizontal transmission, the microsporidian spore is ingested by the silkworm larva through contaminated mulberry leaves and then germinates under alkaline conditions inside the larval midgut [3, 4]. The microsporidian spore sends out a polar tube, which penetrates the epithelial cell and surrounding muscles and spreads out to every part of the larval body. The sporoplasm multiplies by fission, spreads through the hemolymph and reaches every part of the silkworm body such as fat body, muscular tissue, silk gland, and reproductive system [5]. The spore completes its multiplication and life cycle within 4 days. The *N. bombycis* infection may be highly virulent resulting in heavy loss for sericulture practice or result in chronic infection [6]. However, pebrine disease may manifest itself in all stages. As a chronic infection, the infected silkworm larvae survive into the pupal and adult stages and the *N. bombycis* is then vertically transmitted to the eggs [2]. In larvae, the typical symptoms of pebrine are dark spots on the larval integument, and pale, dull, translucent and wrinkled skin [7, 8]. The black spot symptom is a result of an increase of juvenile hormone during infection, which also leads to small larval size, delayed development and molting problems [9]. Highly infected pupae fail to metamorphose into adult moths. Adults experience irregular moth emergence, clubbed wings and low fecundity. In the case of severe infection, non-hatching and dead eggs are observed. In the case of low infection, the eggs hatch and *N. bombycis* develops at the larval stage and causes death in the latter stages of larvae and in pupae. Since pebrine may be vertically transmitted, it is difficult to eliminate from the silkworm rearing. Instead, options for eliminating the pebrine outbreaks could be to select for tolerant silkworm strains, augment the immune response, or apply a treatment against the *N. bombycis*. One such treatment is application of probiotics and recent data indicate that *Nosema* infection is decreased in *Lactobacillus*-treated honey bees (*Apis mellifera*) [10, 11].

Probiotics used as supplementation have been shown to inhibit and reduce growth of pathogens, particularly in honey bees [12]. Probiotics also produce lactic acid, which decrease pH in the midgut, decrease populations of pathogens, reduce gut inflammation and increase immune function and overall gut health [13]. The most common probiotics used are bacteria in the genera *Lactobacillus* and *Bifidobacterium*. *Lactobacillus* is a genus of Gram-positive, non-sporulated and anaerobic bacteria with positive health effects on the gut [14]. Bermudez-Brito et al*.* [15] demonstrated and summarized six major mechanisms of action of probiotics: (1) enhancement of the epithelial barrier, (2) increased adhesion to intestinal mucosa, (3) inhibition of pathogen adhesion, (4) competitive exclusion of pathogenic microorganisms, (5) production of anti-microorganism substances, and (6) modulation of the immune system. In insects, honey bees are known to harbor several species of lactic acid-producing bacteria, which function as probiotics and induce the innate immune response [16]. In bivoltine *B. mori*, the innate immunity was stimulated by *Lactococcus lactis* [17] and by *Lactobacillus paraplantarum* [18]. Subramanian et al*.* [19] studied antibacterial activity and ecofriendly properties of the probiotic *Streptomyces noursei* for management of silkworm diseases. *S. noursei* increased the endogenous actinomycetes population in the silkworm strains PM and CSR2, but inhibited the growth of other Gram-positive and Gram-negative bacteria [19].

Recently, Yeruva et al. [20] used 16S sequencing to investigate the presence of potential probiotics in *B. mori* and found that one of the dominating genera was *Lactobacillus*. We hypothesized that feeding *Lactobacillus* bacteria could reduce the number of dead silkworm larvae challenged by *N. bombycis* and found significant positive effects.

## Main text

### Materials and methods

#### Silkworm rearing

The commercial Thai hybrid silkworm strain, DokBua was used. It is provided to Thai farmers because of its high silk yield. It also has a short life cycle, which results in a short time to obtain silk products. Moreover, it can be reared all year around because of being polyvoltine [21]. Eggs were provided by the Center of Excellence for Silk Innovation, Mahasarakham University (MSU), Thailand. Silkworm eggs were incubated at 25 °C for 8 days. After the egg color changed from yellow to dark gray, the egg surface was disinfected by soaking eggs into 3% formaldehyde solution for 10 min. Then eggs were soaked in 75% ethylalcohol for 1 min, followed by 1 min in 95% ethylalcohol. The eggs were then returned into the 25 °C incubator and incubation was continued until hatching. Newly hatched larvae of DokBua were reared by feeding on mulberry leaves harvested from the mulberry plantation in the experimental station at MSU under standard rearing conditions at 25–26 °C with relative humidity of 75–80%. The silkworm larvae were fed on mulberry leaves only until they became second instar larvae. From the second instar larvae onwards, larvae were fed according to the feeding plans described in “Feeding experiments” below.

#### Preparation of *Nosema bombycis*

Pebrine-infected silkworm larvae were collected from silkworm household sericulturists in the Borabue village, Mahasarakham province, Thailand in May 2017. Spores of *N. bombycis* were propagated by oral inoculation of 3^rd^ larvae and isolated in the laboratory of MSU. Fifteen diseased larvae were crushed in distilled water and the crude extract was filtered through a fine muslin cloth. The filtrate was transferred into a centrifuge tube and centrifuged in a Sigma 6–16 Centrifuge (Sigma Laborzentrifugen GmbH, Osterode am Harz, Germany) at 4340×*g* for 5 min. The supernatant was discarded and the pellet was suspended in distilled water and washed three times. To confirm the presence of *N. bombycis* spores, the suspension was examined by phase contrast light microscopy at 400× magnification by the first author Siripuk Suraporn. The concentration of spores was counted with a hemocytometer by the first author Siripuk Suraporn. The *N. bombycis* spores were fed to the silkworm larvae at a concentration of 10^6^ spores/mL.

#### Preparation of *L. casei*

The *Lactobacillus casei* strain TISTR 1463 was provided from the Thailand Institute of Scientific and Technological Research (TISTR). Pure cultures were obtained by streaking out bacteria on Luria Broth (LB) agar (2% NaCl; Difco, Lawrence, KS, USA) and incubated at room temperature overnight. A single colony was picked up and transferred into 10 mL of LB broth, supplemented with 2% NaCl (w/v) and incubated at 28 °C overnight with agitation. The *L. casei* culture was transferred into 50 mL of Man, Rogosa, Sharpe (MRS; Difco, Lawrence, KS, USA) medium broth and incubated under the same conditions overnight. The culture broth was then centrifuged at 4340×*g* at 4 °C for 15 min. The pellet of *L. casei* was collected and washed with distilled water. The concentration of the culture suspension used to feed the silkworm larvae (10^8^ bacteria/mL) was determined by counting with a hemocytometer (Boeco, Hamburg, Germany).

#### Feeding experiments

To explore if *L. casei* had a protective effect on *Nosema* infection, *B. mori* larvae were fed on mulberry leaves cut in pieces of 2 × 2 cm and dipped in *L. casei* (10^8^ bacteria/mL), followed by mulberry leaves dipped in *N. bombycis* (10^6^ spores/mL). Three control treatments were set up: (1) larvae fed on mulberry leaves only, (2) larvae fed on mulberry leaves supplemented with *L. casei* (10^8^ bacteria/mL), (3) larvae fed on mulberry leaves supplemented with *N. bombycis* (10^6^ spores/mL). Two mL of *L. casei* or parasite solution were used per 100 larvae and the larvae consumed all solution provided. Mulberry leaves supplemented with *L. casei* were given on the second day after molting in the 2nd, 3rd, 4th and the 5th instar (thus at day 5, 8, 12 and 16). Mulberry leaves supplemented with *N. bombycis* spores were given on the second day after molting in the 4th instar. Each experiment was replicated three times with 25 silkworm larvae per replication. The number of deceased larvae were counted until the end of the 5th instar. Also measured were larval weight of the 5th instar, cocooning ratio, pupation ratio, cocoon weight, and cocoon shell weight. The data were analyzed using Duncan multiple range test at 95% confidence level with Statistica 4.3.

### Results

#### Pebrine infection

**Fig. 1:**
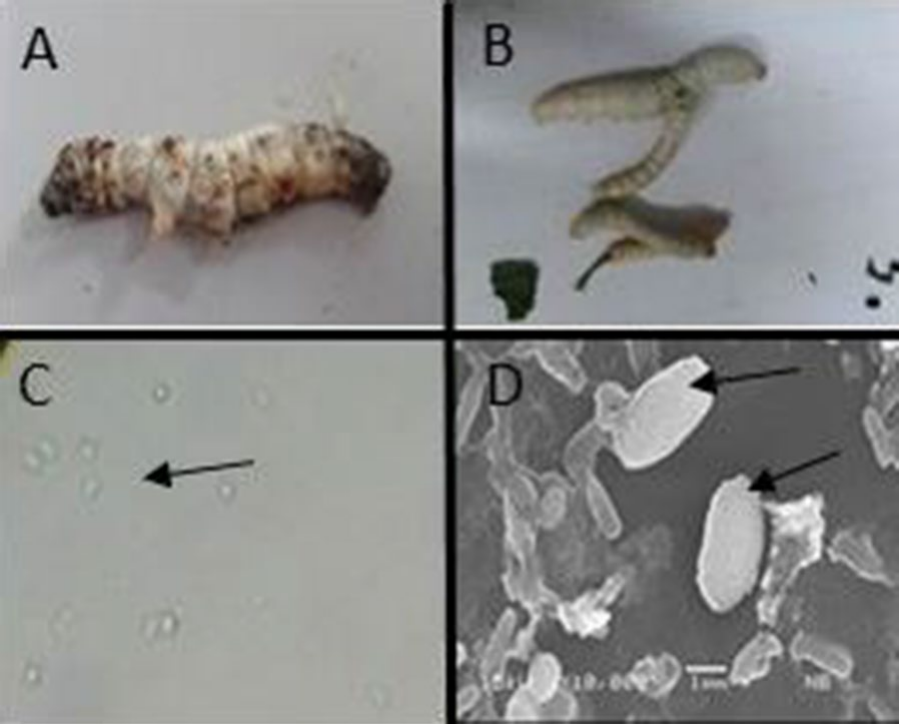
The symptom of pebrine by *Nosema bombycis*, **a** so-called black pepper symptom found in the 5th instar larva, **b** size differences within the same instar, **c**, **d** spores of *Nosema bombycis* observed under light microscope (40X magnification) and scanning electron microscope, respectively

Severely infected silkworm larvae had spotted markings, which could be seen within 8 days after infection (Fig. 1a). The larvae displayed unequal body size in the same instar due to retarded growth of diseased larvae (Fig. 1b). Other symptoms were slow motion and no feeding, which was observed 5 days after infection. *N. bombycis* spores in infected larvae were oval and reflective (Fig. 1c, d).

**Fig. 2:**
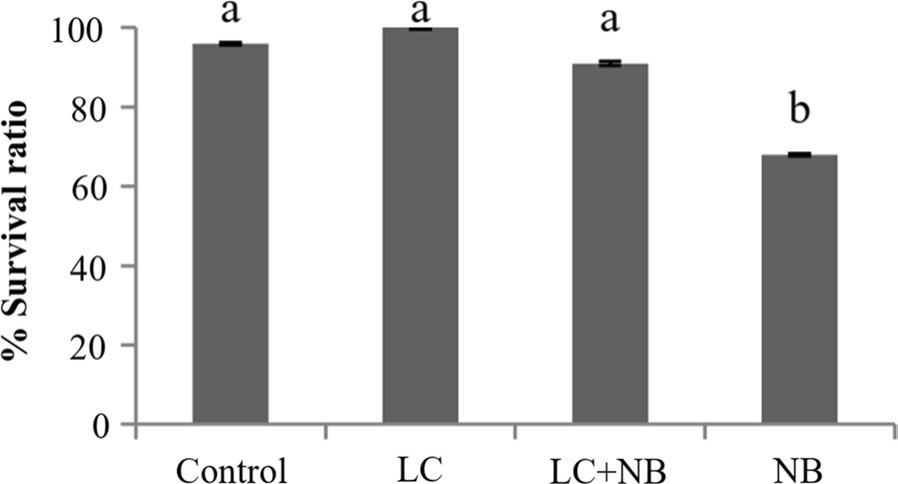
The effects of *Lactobacillus casei* (LC) and *Nosema bombycis* (NB) on survival ratio of *B. mori* larvae. Treatments were performed with and without 10^8^ cells/mL of *L. casei* and 10^6^ spores/mL of *N. bombycis* and the results were compared to those of the control group, which were fed with distilled water. Different letters on the top of each bar represent difference identified by the Analysis of Variance coupled with Duncan’s Multiple Range Test at 95% significant level. Bars represent 1 unit of standard deviation

The feeding experiments resulted in a statistically significant difference in survival ratio for pebrine-infected larvae supplemented with *L. casei* (91.00 ± 0.57%) as compared to pebrine-infected larvae not receiving *L. casei* (68.00 ± 0.33%; Fig. 2). There was no statistical difference in survival ratio for silkworm larvae supplemented with *L. casei* (100.00 ± 0.33%), control larvae (96.00 ± 0.33), and larvae receiving both *L. casei* and *N. bombycis* (91.00 ± 0.33%). For the silkworm larvae surviving until pupation, growth characters such as larval weight of the 5th instar (g), cocooning ratio (%), and pupation ratio (%), and economic characters such as cocoon weight (g) and cocoon shell weight (g) were determined (Table 1).

**Table 1:** Effects of *Lactobacillus casei* and *Nosema bombycis* challenge on Thai polyvoltine strain, DokBua, of the silkworm, *Bombyx mori*

Treatments (n = 75)	Quality parameter (means ± SD)				
	Growth characters			Economic characters	
	Larval weight of 5th instar (g)	Cocooning ratio (%)	Pupation ratio (%)	Cocoon weight (g)	Cocoon shell weight (g)
*Lactobacillus casei* (10^8^ cell/mL) followed by *Nosema bombycis* (10^6^ spores/mL)	2.70 ± 0.96^a^	95.00 ± 0.00^ab^	95.00 ± 0.33^a^	1.33 ± 0.33^a^	0.23 ± 0.33^a^
No treatment	2.55 ± 0.48^a^	96.00 ± 0.33^b^	100.00 ± 0.33^b^	1.23 ± 0.67^a^	0.20 ± 0.57^a^
*Lactobacillus casei* (10^8^ cells/mL)	2.84 ± 0.48^a^	96.00 ± 0.57^b^	100.00 ± 0.57^b^	1.40 ± 0.58^a^	0.23 ± 0.67^a^
*Nosema bombycis* (10^6^ spores/mL)	2.12 ± 0.96^b^	93.00 ± 0.33^a^	87.00 ± 0.66^c^	1.27 ± 0.33^a^	0.23 ± 0.33^a^

Statistically significant differences as measured by Duncan multiple range test are denoted with different letters

The *L. casei* supplementation had positive impact on the growth characters (Table 1). As compared to larvae receiving only *N. bombycis*, the 5^th^ larval instar weight was significantly higher in all groups. *N. bombycis* infection significantly lowered the cocooning ratio, but *L. casei* treatment made the ratio remain on an intermediate level. The pupation ratio was 100% in the groups not infected by *N. bombycis*. The *L. casei* treatment resulted in a significantly higher pupation ratio in *N. bombycis*-infected larvae (95% with *L. casei* vs. 87% without *L. casei*). The highest cocoon weight and cocoon shell weight were obtained in those larvae receiving *L. casei*, although this difference was not statistically significant.

### Discussion

A major problem for the silk production in Thailand is diseases of silkworm larvae. The microsporidian *Nosema bombycis*, which leads to the chronic disease pebrine, is a key pathogen for Thai polyvoltine silkworm strains and causes severe economic loss in sericulture. In this paper, we explored the possibility to treat *Bombyx mori* larvae with *Lactobacillus casei* to mitigate the effect of *N. bombycis* infection. Our study demonstrated that supplementation of *L. casei* at a concentration of 10^8^ bacteria/mL made pebrine-infected silkworm larvae survive at the same level as non-infected larvae.

The midgut environment of the silkworm larva plays a major role in the resistance/tolerance or susceptibility to pathogens including *N. bombycis*. The highest germination of *Nosema* occurs at pH 9–10 [6, 22]. Thus, the decrease in pebrine mortality in larvae treated with lactic acid bacteria could be related to a reduction of pH in the midgut, which possibly led to lowered infectivity of the spores. An alternative mechanism of action that could affect the *N. bombycis* infection might be a competitive adhesion of *L. casei* to the intestinal mucosa, which would inhibit pathogen adhesion. Potentially, modulation of the immune system by *L. casei* could lead to production of anti-microbial substances [17]. Indeed, Nishida et al*.* [12] demonstrated that the lactic acid bacteria *Lactococcus lactis* stimulated the innate immunity in silkworms.

We also showed that the *L. casei* treatment led to increased weight gain in the DokBua polyvoltine silkworm strain. Although not statistically different, the average weight of the 5th larval instar was higher in larvae receiving *L. casei* as compared to control. This was true also for *N. bombycis*-infected larvae receiving *L. casei* whereas infected larvae without *L. casei* treatment had a significant weight loss as compared to all other groups. Thus, the *L. casei* treatment seemed to increase food digestion and absorption of nutrients, which promoted growth characters. Similarly, Sekar et al*.* [23] studied the effect of several species of *Bacillus* and *Lactobacillus* on the growth parameters and cocoon production in *B. mori* by adding putative probiotic strains as supplementation with mulberry leaves. They found that *L. casei* enhanced length and weight of the larval body, and significantly increased silkworm cocoon weight. Alternatively, or in addition, *L. casei* may have increased the protection against harmful bacteria, thus potentially leading to resource allocation towards growth rather than immune response.

## Limitations

This is a preliminary proof-of-principle showing that supplementation of *Lactobacillus* results in reduced mortality for silkworm larvae challenged by *Nosema bombycis*. Further studies are needed to elucidate the role and mechanism of this effect and to optimize the application of bacteria. For example, is the protective effect caused by a lower pH, and if so, for how long does it remain after supplementation of bacteria? May the microsporidian gene expression be affected by the presence of *L. casei*? Will treatment with *Lactobacillus* lead to complete extermination of microsporidia, or may a fraction remain that in turn could cause vertical transmission by surviving pupae? While the study shows an effect on *N. bombycis* that has been orally infected, there is also a vertical transmission of these parasites, does the supplementation of *L. casei* have any effect on vertically transmitted microsporidia? How much bacteria are needed and when should they be applied for optimal results?

This preliminary study has been conducted with only one concentration of bacteria and one concentration of *Nosema*. It has also focused on one strain of bacteria, parasite and host. Changing any of these five parameters may change the outcome.

## Data Availability

The datasets generated during and/or analyzed during the current study are available from the corresponding author on reasonable request.

## Abbreviations

MSU: Mahasarakham University
TISTR: Thailand Institute of Scientific and Technological Research
LB: Luria Broth
MRS: Man, Rogosa, Sharpe

## References

[1] Bhat SA, Bashir I, Kamili AS (2009). Microsporidiosis of silkworm, *Bombyx mori* L. (Lepidoptera-Bombycidae): a review. Afr J Agric Res.

[2] Han MS, Watanabe H (1988). Transovarial transmission of two microsporidia in the silkworm, *Bombyx mori*, and disease occurrence in the progeny population. J Invertebr Pathol.

[3] Peter A, Sadatulla F, Devaiah MC, Devaiah MC, Narayanaswamy KC, Maribashetty VG (1999). The viral, bacterial and protozoan diseases of the silkworm, *Bombyx mori* L. Advance in mulberry sericulture.

[4] Franzen C (2005). How do microsporidia invade cells?. Folia Parasitol.

[5] Fujiwara T (1985). Microsporidia from silkworm moths in egg production sericulture. J Seric Sci.

[6] Singh T, Bhat MM, Khan MA (2012). Microsporidiosis in the silkworm, *Bombyx mori* L. (Lepidoptera: Bombycidae). Pertanika J Trop Agric Sci.

[7] Singh T, Saratchandra B (2003). Microsporidian disease of the silkworm, *Bombyx mori* L. (Lepidoptera: Bombycidae). Int J Indust Entomol.

[8] Solter LF, Becnel JJ, Oi DH, Vega FE, Kaya HK (2012). Microsporidian Entomopathogens. Insect pathology (second editor).

[9] Szumowski SC, Troemel R (2015). Microsporidia-host interactions. Curr Opin Microbiol.

[10] Arredondo D, Castelli L, Porrini MP, Garrido PM, Eguaras MJ, Zunino P, Antúnez K (2018). *Lactobacillus kunkeei* strains decreased the infection by honey bee pathogens *Paenibacillus larvae* and *Nosema ceranae*. Benef Microbes.

[11] Tejerina MR, Benítez-Ahrendts MR, Audisio MC (2020). *Lactobacillus salivarius* A3iob reduces the incidence of *Varroa destructor* and *Nosema* spp. in commercial apiaries located in the northwest of Argentina. Probiot Antimicrob Proteins.

[12] Nishida S, Ono Y, Sekimizu K (2016). Lactic acid bacteria activating immunity improve survival in bacterial infection model of silkworm. Drug Discov Ther.

[13] Nishida S, Nishiya Y, Abe S, Ono Y, Sekimizu K (2017). *Lactobacillus paraplantarum* 11–1 isolate from rice bran pickles activated innate immunity and improved survival in a silkworm bacterial infection model. Front Microbiol.

[14] Hamdi C, Balloi A, Essanaa J, Crotti E, Gonella E, Raddadi N, Ricci I, Boudabous A, Borin S, Manino A, Bandi C, Alma A, Daffonchio D, Cherif A (2011). Gut microbiome dysbiosis and honey bee health. J Appl Entomol.

[15] Ramos OY, Basualdo M, Libonatti C, Vega MF (2020). Current status and application of lactic acid bacteria in animal production systems with a focus on bacteria from honey bee colonies. J Appl Microbiol.

[16] Walker WA (2008). Mechanism of action of probiotics. Clin Infect Dis (CID).

[17] Bermudez-Brito M, Diaz JP, Quezada SM, Llorente CG, Gil A (2012). Probiotic mechanisms of action. Ann Nutr Metab.

[18] Evans JD, Lopez DL (2004). Bacterial probiotics induce and immune response in the honey bee (Hymenoptera:Apidae). J Econ Entomol.

[19] Subramanian S, Mohanrag P, Muthuswamy M (2009). New paradigm in silkworm disease management using probiotic application of *Streptomyces noursei*. Karnataka J of Agric Sci.

[20] Yeruva T, Vankadara S, Ramasamy S, Lingaiah K (2020). Identification of potential probiotics in the midgut of mulberry silkworm, *Bombyx mori* through metagenomic approach. Probiot Antimicrob Proteins.

[21] The Queen Sirikit Department of Sericulture. The Thai hybrid silkworm rearing in Thailand. In: Proceeding of the 24th congress on sericulture and silk industry, Bangkok, Thailand, 10–14 August 2016; 2016.

[22] Undeen AH, Epsky ND (1990). In vitro and in vivo germination of *Nosema locustae* (Microspora: Nosematidae) spores. J Invert Pathol.

[23] Sekar P, Kalpana S, Ganga S, John G, Kannadasan N (2016). Effect of the probionts to the enhancement of silk proteins (sericin and fribroin) in the silk gland and cocoons of silkworm (LxCSR2) *Bombyx mori* (L.). Int J Pharm Biol Sci.

